# Gene Expression Pattern in Transmitochondrial Cytoplasmic Hybrid Cells Harboring Type 2 Diabetes-Associated Mitochondrial DNA Haplogroups

**DOI:** 10.1371/journal.pone.0022116

**Published:** 2011-07-13

**Authors:** Seungwoo Hwang, Soo Heon Kwak, Jong Bhak, Hae Sun Kang, You Ri Lee, Bo Kyung Koo, Kyong Soo Park, Hong Kyu Lee, Young Min Cho

**Affiliations:** 1 Korean Bioinformation Center, Korea Research Institute of Bioscience and Biotechnology, Daejeon, Korea; 2 Department of Internal Medicine, Seoul National University College of Medicine, Seoul, Korea; 3 Theragen BiO Institute, Suwon, Korea; 4 Department of Internal Medicine, Eulji University College of Medicine, Seoul, Korea; University of California Los Angeles, United States of America

## Abstract

Decreased mitochondrial function plays a pivotal role in the pathogenesis of type 2 diabetes mellitus (T2DM). Recently, it was reported that mitochondrial DNA (mtDNA) haplogroups confer genetic susceptibility to T2DM in Koreans and Japanese. Particularly, mtDNA haplogroup N9a is associated with a decreased risk of T2DM, whereas haplogroups D5 and F are associated with an increased risk. To examine functional consequences of these haplogroups without being confounded by the heterogeneous nuclear genomic backgrounds of different subjects, we constructed transmitochondrial cytoplasmic hybrid (cybrid) cells harboring each of the three haplogroups (N9a, D5, and F) in a background of a shared nuclear genome. We compared the functional consequences of the three haplogroups using cell-based assays and gene expression microarrays. Cell-based assays did not detect differences in mitochondrial functions among the haplogroups in terms of ATP generation, reactive oxygen species production, mitochondrial membrane potential, and cellular dehydrogenase activity. However, differential expression and clustering analyses of microarray data revealed that the three haplogroups exhibit a distinctive nuclear gene expression pattern that correlates with their susceptibility to T2DM. Pathway analysis of microarray data identified several differentially regulated metabolic pathways. Notably, compared to the T2DM-resistant haplogroup N9a, the T2DM-susceptible haplogroup F showed down-regulation of oxidative phosphorylation and up-regulation of glycolysis. These results suggest that variations in mtDNA can affect the expression of nuclear genes regulating mitochondrial functions or cellular energetics. Given that impaired mitochondrial function caused by T2DM-associated mtDNA haplogroups is compensated by the nuclear genome, we speculate that defective nuclear compensation, under certain circumstances, might lead to the development of T2DM.

## Introduction

Mitochondrial dysfunction plays a key role in the pathogenesis of insulin resistance and pancreatic β-cell dysfunction, the two major pathophysiological defects of type 2 diabetes mellitus (T2DM) [1], [2]. The glucose-stimulated insulin secretion in pancreatic β-cells is triggered by the closure of ATP-sensitive potassium channels, which is dependent on intracellular ATP levels generated from mitochondria [3]. In this regard, it is of note that the pancreatic β-cell-specific knockout of mitochondrial transcription factor A results in diabetes due to severe impairment of insulin secretion and β-cell loss in mice [4]. Insulin resistant offspring of T2DM patients revealed an approximately 60% decrease in insulin-stimulated glucose uptake and an approximately 30% decrease in mitochondrial oxidative phosphorylation (OXPHOS) capacity in the skeletal muscle [1], implying that decreased mitochondrial function may also lead to skeletal muscle insulin resistance. Genome-wide expression analyses have demonstrated that genes involved in mitochondrial OXPHOS were coordinately down-regulated in the skeletal muscle of T2DM subjects presumably due to decreased PGC-1α (peroxisome proliferator-activated receptor γ coactivator 1α) expression, which is a key regulator of mitochondrial function and biogenesis [5], [6]. Therefore, it is essential to figure out the causes of mitochondrial dysfunction to prevent and treat T2DM.

Genetic variations in nuclear DNA or mitochondrial DNA are known to be associated with decreased β-cell function and/or the risk of T2DM[5], [6], [7], [8], [9], [10]. An A to G substitution at nucleotide position 3243 (A3243G) of mtDNA, which encodes leucyl-tRNA^UUR^, is commonly associated with maternally inherited diabetes and deafness [3], [11]. A common mtDNA polymorphism, T16189C, is significantly associated with T2DM at least in Asians, including Koreans, Japanese, and Chinese, with an odds ratio of 1.26 (95% confidence interval 1.08–1.46) [7]. The binding affinity of the mitochondrial single-stranded binding protein is altered by the T16189C variant [7], implying that this variant may alter mitochondrial function. However, the association between the T16189C variation and T2DM was not evident in Europeans [12]. Ethnic differences in nuclear and mitochondrial genomic background as well as environmental factors may explain this discrepancy. Interestingly, geographic region-specific mtDNA haplogroups are thought to modify mitochondrial function because there is evidence that they are formed by adaptation to thermal environments and subsequent natural selection [13], [14]. Contrary to this notion, it was reported that mtDNA haplogroups were not associated with the risk of T2DM in Europeans [15]. However, it was recently demonstrated that mtDNA haplogroups are associated with susceptibility to T2DM in both Koreans and Japanese [10]. Among the 10 most common mtDNA haplogroups in Koreans and Japanese, the N9a haplogroup was significantly associated with a decreased risk of T2DM, while haplogroups D5 and F were associated with an increased risk of T2DM [10]. However, the mechanisms by which mtDNA haplogroups N9a, D5, and F modulate the susceptibility to T2DM still remain elusive. In this regard, it is noteworthy that conplastic strains of rats with identical nuclear genomes but divergent mitochondrial genomes display significant differences in glucose and insulin response after oral glucose load [16], implying that the mitochondrial genome *per se* (or a certain mtDNA haplogroup) may affect metabolic phenotypes.

**Figure 1:**
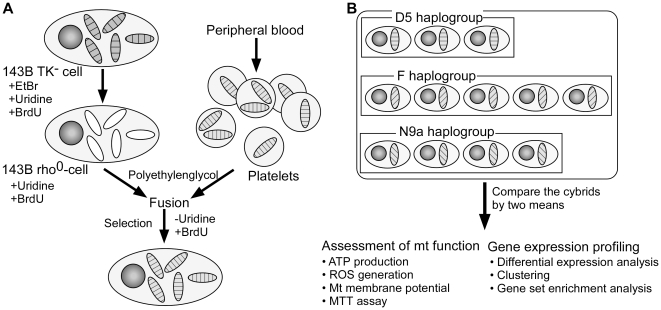
Schematic of cybrid generation and data analysis. (A) Generation of cybrids. Round circle: nucleus, filled ovals: intact mitochondria, unfilled ovals: mtDNA-depleted mitochondria. (B) Comparison of cybrids originating from three mtDNA haplogroups.

To examine functional differences of mtDNA haplogroups independent of different nuclear genomes from individual subjects, we took advantage of the transmitochondrial cytoplasmic hybrid (cybrid) model (Figure 1), which is generated by the fusion of mtDNA-depleted cells (rho^0^) with mtDNA donors (either enucleated cells or platelets) [17]. Platelets, which are formed by the cytoplasmic budding of megakaryocytes, are suitable as mtDNA donors because they lack nuclei but contain intact mitochondria [18]. The merit of using an identical rho^0^ cell line as the mtDNA recipient is to avoid the confounding effects of having different nuclear genomes from different subjects, which is a major hurdle when studying tissues from different individuals [19]. Thus, the cybrid model enables us to isolate the functional consequences of different mtDNA haplogroups, including altered mitochondrial function and/or differential expression of nuclear genes induced by retrograde signaling from mitochondria to nucleus. In the present study, we compared functional characteristics of cybrid cells harboring T2DM-resistant haplogroup N9a or T2DM-susceptible haplogroups D5 and F with cell-based functional assays. In addition, we performed microarray analyses to examine differences in gene expression that might arise from the mitochondria-nuclear interaction.

## Materials and Methods

### Generation of cybrid cells

The Institutional Review Board of the Clinical Research Institute at Seoul National University Hospital approved the study protocol, and written informed consent was obtained from each subject. To produce cybrid clones, we used platelets from 12 non-diabetic subjects harboring haplogroups D5 (n = 3), F (n = 5), and N9a (n = 4), selected from our previous study [10]. The osteosarcoma cell line with neither mtDNA nor thymidine kinase activity (143B TK^−^ ρ^0^) was generously provided by Professor Yau-huei Wei from National Yang-Ming University, Taipei, Taiwan. It was obtained after long-term exposure to ethidium bromide (50 ng/ml) and was grown in Dulbecco's modified Eagle's medium (DMEM) supplemented with 100 mg/ml 5-bromodeoxyuridine, 50 µg/ml uridine, and 10% fetal bovine serum. Southern blot analysis and PCR amplification of the mtDNA target sequences confirmed the absence of any residual mtDNA (data not shown). Using platelets as mtDNA donors, cybrid cells were produced as described previously [18] (Figure 1). Briefly, the platelet-rich fraction was separated by centrifugation from venous whole blood and fused to 143B TK^−^ ρ^0^ cells in the presence of 42% polyethylene glycol 1500 (Sigma-Aldrich, St. Louis, MO). Sixty seconds after the addition of 42% polyethylene glycol, the cells were resuspended in DMEM. After twenty-four hours, the medium was replaced with DMEM containing 5-bromodeoxyuridine but lacking uridine to select only successfully fused cells. The reconstitution of mtDNA haplogroups in cybrid cells was confirmed by restriction fragment length polymorphism specific to each mtDNA haplogroup (data not shown). To assure a complete repopulation of mtDNA haplogroups, cell-based functional assays and microarray experiments were carried out after 2–3 months of successive subcultivation.

### Cell-based functional measurements

The detailed methods for cell-based functional assays are provided in the File S2. In brief, cybrid cells were seeded in 96-well plates at a density of 1×10^6^ cells/ml. After stabilization for 24 hours at 37°C under 5% CO_2_/95% air (vol/vol), each functional assay was performed using Victor3 multilabel plate reader (PerkinElmer, Waltham, MA). Cellular ATP levels were measured using the ATPLite kit (PerkinElmer) and reactive oxygen species (ROS) were measured using chloromethyl-2′7′-dichlorodihydrofluorescein diacetate acetyl ester (CM-H2DCFDA) (Sigma-Aldrich, St. Louis, MO). Mitochondrial membrane potential (MMP) was measured using 5,5′,6,6′-tetrachloro-1,1′,3,3′-tetraethylbenzimidazolylcarbocyanine iodide (JC-1) dye (Molecular Probes, Carlsbad, CA) and cellular viability and mitochondrial dehydrogenase activity were measured by the 3-(4,5-dimethylthiazol-2-yl)-2,5-diphenyltetrazolium bromide (MTT) (Sigma-Aldrich, St. Louis, MO) reduction assay.

### Statistical analysis of functional measurements

Data are expressed as the mean ± SE. Significant differences between groups were evaluated using the Mann-Whitney test. Differences were considered statistically significant when *p*-values were <0.05. Statistical analyses were performed using SPSS 12.0 software for Windows (SPSS Inc., Chicago, IL).

### Microarray experiments

Isolation of total RNA from cells was performed using the RNeasy Micro Kit (Qiagen, Valencia, CA) according to the manufacturer's instructions. Targets for the GeneChip Human Gene 1.0 ST Array (Affymetrix, Santa Clara, CA) were prepared and hybridized with the GeneChip Whole Transcript Sense Target Labeling Assay (Affymetrix) and scanned according to the manufacturer's instructions (Scanner 3000 7G, Affymetrix). The data of each cybrid were acquired in four replicates. The microarray data were all MIAME compliant and were deposited to Gene Expression Omnibus under the accession number GSE26244.

### Microarray data preprocessing

The CEL files were imported to the Affymetrix Expression Console software and normalized by the Robust Multi-chip Average method [20] and log_2_-transformed. Expression data from probes without gene symbol annotations (based on NetAffx annotation accessed in November 2008) were discarded. Expression levels from multiple probes that represent the same gene were averaged into a single value within each array. Expression data from four technical replicates from each cybrid were also averaged, yielding gene-level averaged and replicate-averaged expression profiles for 20,196 unique genes and 12 cybrid samples, which were used for all subsequent analyses.

### Functional enrichment analysis

Functional enrichment analysis was performed using two independent methods, gene set enrichment analysis (GSEA) [21] and GeneTrail [22], to provide more confidence in the findings. Both of them have advantages in that they can identify functional categories with subtle but coherent expression changes by bypassing a cutoff-dependent identification step of differentially expressed genes. To prepare input data for the functional enrichment analysis, a sorted list of all genes ranked by Welch *t*-statistic was made for each of three pairwise haplogroup comparisons. Enrichment analyses were calculated under the pre-ranked mode with 1,000 permutations on GSEA and the gene set mode on GeneTrail. Since the focus of this study is to identify metabolic differences between haplogroups, GSEA and GeneTrail analyses were run against KEGG metabolic pathways only. The minimum and maximum sizes of gene sets were set to 25 and 500, respectively, in both analyses. A pathway was considered significantly regulated if its *p*-value was <0.05 and the false discovery rate was <0.25, as has generally been done [6].

### Differential expression and clustering analysis

To simplify the biological interpretation of results from differential expression and clustering analysis, only the data from probes that represent well-characterized genes were used in the analysis by retaining only the protein-coding or mitochondrially encoded gene probes that are annotated with the gene symbol and gene ontology biological process. There were a total of 13,379 such genes.

To identify genes that are differentially expressed between haplogroups, analysis of variance (ANOVA) followed by Tukey's pairwise comparison was performed using R/Bioconductor. Genes were considered significantly differentially expressed if *p*-values from ANOVA and Tukey's test were both below 0.05; a total of 2,361 genes met these criteria. Among them, 210 genes also satisfied a minimum criterion of a 1.2-fold change between haplogroup pairs.

Hierarchical clustering analysis was performed using two complementary methods: a usual gene-based clustering and a gene set-based clustering. The gene-based clustering that groups genes and samples was done using the Genesis [23] software with expression profile data of 210 differentially expressed genes that satisfy both statistical and fold change criteria. The gene set-based clustering that groups gene sets and samples was done on the PathCluster [24] software with expression profile data of 2,361 differentially expressed genes that satisfy statistical significance only. In accordance with the focus of this study, PathCluster analysis was run against KEGG metabolic pathways only. The minimum gene set size was set to 3. In both clustering analyses, the expression profile of each gene was standardized with a z-transformation such that the row mean becomes 0 and the row variance becomes 1, and hierarchical clustering was done using average linkage clustering with Pearson centered correlation as a similarity metric.

## Results

### Mitochondrial functions in cybrid cells harboring different mtDNA haplogroups

**Figure 2:**
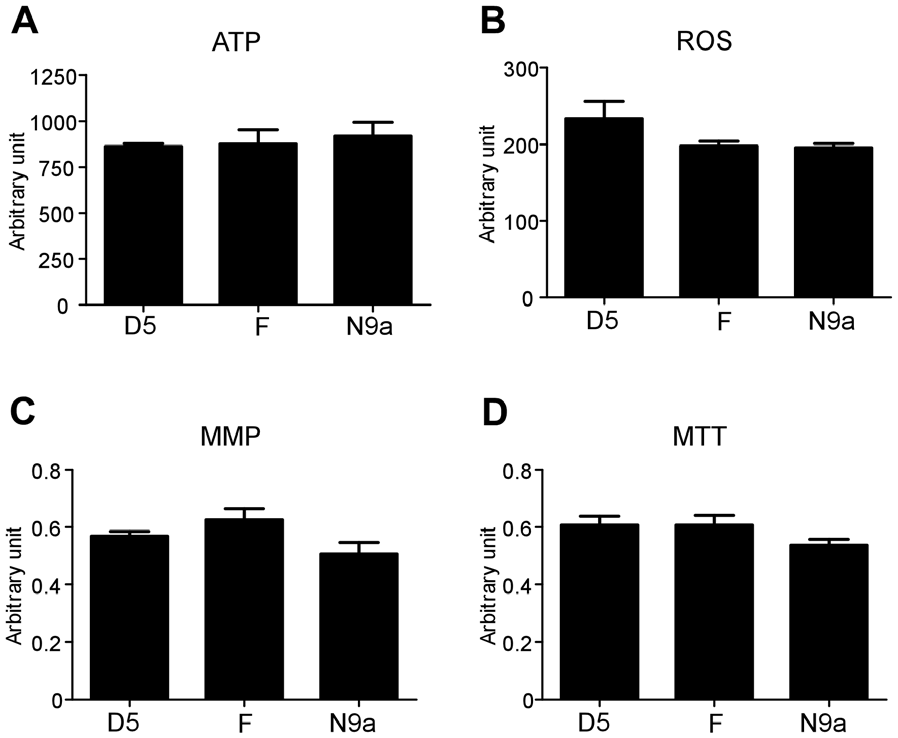
Assessment of mitochondrial function of cybrids harboring three mtDNA haplogroups. ATP, ROS, MMP, and MTT assays were performed. Each experiment was repeated five times. Data are presented as the mean ± SE.

We examined the mitochondrial functions of cybrids harboring mtDNA haplogroups D5, F, and N9a. Mitochondrial function was assessed in terms of ATP production, ROS generation, MMP, and cellular dehydrogenase activity. There were no significant differences in these assays among the three haplogroups examined (Figure 2).

### Haplogroup comparison by differential expression analysis

**Figure 3:**
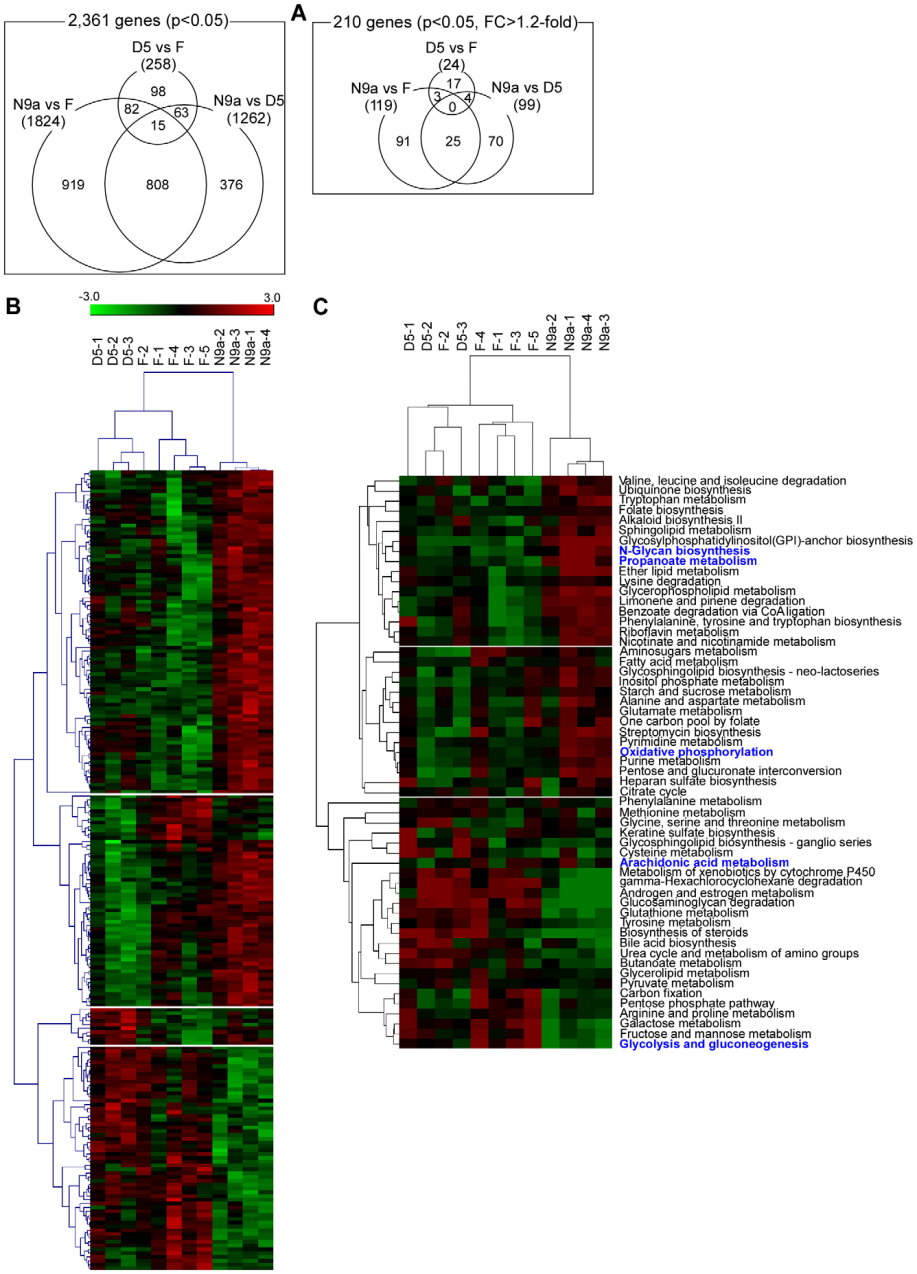
Results from differential expression and clustering analyses. (**A**) Three-way Venn diagrams that show the number of differentially expressed genes that are in common or specific to each of the amongthree mtDNA haplogroups. Left—Venn diagram of 2,361 genes that were statistically significant; Right—Venn diagram of 210 genes with more than 1.2-fold pairwise change as well as being statistically significant. (**B**) Hierarchical clustering of 12 cybrids using the expression profile of 210 genes. (**C**) Metabolic pathway-based hierarchical clustering of 12 cybrids using PathCluster. Five pathways in blue indicate the metabolic pathways in Table 1 whose differential regulation were found to be statistically significant by GSEA analysis.

We examined differential gene expression among the three haplogroups. A total of 2,361 genes met the *p*-value cutoff <0.05 for the ANOVA and Tukey's test. Among them, 210 genes satisfied the pairwise fold-change criterion >1.2-fold (listed in File S1). Figure 3A shows the number of differentially expressed genes and their overlap, where the smallest was found in the D5 vs. F comparison, suggesting a similarity in the gene expression signature between haplogroups D5 and F.

### Clustering of expression profile

The expression profile of the 210 genes obtained above was subjected to hierarchical clustering (Figure 3B). All samples but F-2 were clustered according to their haplogroup assignments. Haplogroups D5 and F were grouped together, with haplogroup N9a being separated by a large inter-cluster branch length. Next, we analyzed the expression profile of 2,361 genes from the ANOVA analysis by a metabolic pathway-based hierarchical clustering using PathCluster (Figure 3C), which showed a similar pattern of clustering to that shown in Figure 3B.

### Haplogroup comparison by gene set enrichment analysis on metabolic pathways

We compared gene expression levels of metabolic pathways between the three mtDNA haplogroups using GSEA, which reveals coordinated transcriptomic alterations of gene sets [21]. Several metabolic pathways showed differential expression between the three mtDNA haplogroups (Table 1). When haplogroups D5 and F were compared, only the N-glycan biosynthesis pathway showed differential expression. When haplogroups N9a and D5 were compared, only the arachidonic acid metabolism pathways were differentially expressed. In the comparison of N9a and F, haplogroup F showed up-regulation of glycolysis/gluconeogenesis and down-regulation of three pathways: N-glycan biosynthesis, propanoate metabolism, and OXPHOS. The up-regulation of the glycolysis/gluconeogenesis pathway is thought to be attributable to glycolysis since the expression levels of key enzymes in gluconeogenesis (PC, PCK1, PCK2, FBP1, FBP2, and G6PC) were insignificant with a fold-change range of 1.02–1.13 and a *p*-value range of 0.07–0.59. To further substantiate the GSEA analysis result, we also repeated the analysis using GeneTrail [22], which is similar to GSEA in that it is also a cutoff-free enrichment analysis method. The GeneTrail result was similar to the GSEA result (Table S2). In particular, all metabolic pathways in Table 1 were also found to be significantly regulated by GeneTrail, with even smaller *p*-values.

**Table 1:** Significant differentially expressed metabolic pathways identified from haplogroup comparisons using GSEA.

Comparison	Pathway ID	Pathway name	NES	Nominal*p*-value	FDR*q*-value
D5 vs. F	Less active in D5 (More active in F)				
	None				
	More active in D5 (Less active in F)				
	HSA00510	N-Glycan biosynthesis	1.99	0.000	0.007
N9a vs. D5	Less active in N9a (More active in D5)				
	HSA00590	Arachidonic acid metabolism	−1.42	0.049	0.235
	More active in N9a (Less active in D5)				
	None				
N9a vs. F	Less active in N9a (More active in F)				
	HSA00010	Glycolysis and gluconeogenesis	−1.59	0.002	0.230
	More active in N9a (Less active in F)				
	HSA00510	N-Glycan biosynthesis	1.92	0.000	0.015
	HSA00640	Propanoate metabolism	1.53	0.023	0.213
	HSA00190	Oxidative phosphorylation	1.46	0.014	0.249

Criteria for significance are nominal *p*-value <0.05 and FDR *q*-value <0.25. Core sets of genes for these pathways are listed in Table S1. Abbreviations: NES—normalized enrichment score, FDR—false discovery rate.

**Figure 4:**
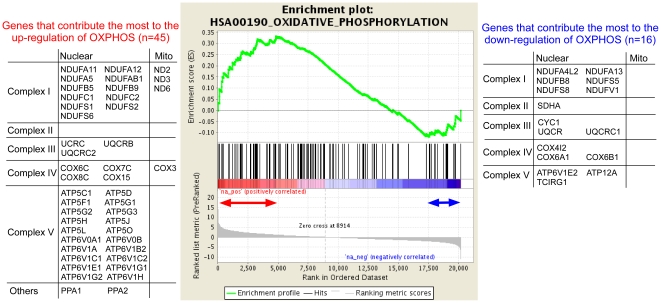
GSEA enrichment plot for oxidative phosphorylation in the comparison of N9a vs. F haplogroups. The plot shows that OXPHOS pathway is overall more active in haplogroup N9a than in haplogroup F. Each of the black peaks represent a gene in the OXPHOS pathway. There are a total of 125 OXPHOS genes. The 45 genes within the red double-headed arrow are the genes that contribute the most to the overall up-regulation of OXPHOS. Note that OXPHOS is also down-regulated to some degree. The 16 genes within the blue double-headed arrow are down-regulated the most.

To see whether nuclear genes or mitochondrial genes were responsible for the observed up-regulation of OXPHOS in haplogroup N9a under its comparison with haplogroup F, we present the list of OXPHOS genes in the leading-edge subset (the core set of genes that contribute the most to the differential regulation of the pathway) (Figure 4) [21]. As shown in Figure 4, both nuclear and mitochondrial genes (45 genes) were responsible for the up-regulation of OXPHOS. In addition, the OXPHOS pathway showed a small down-regulation driven by 16 nuclear genes, which may be due to a homeostatic response in which the up-regulation of one component in a pathway leads to the down-regulation of another component in that pathway in an attempt to compensate [25].

## Discussion

In this study, we investigated functional consequences of three mtDNA haplogroups (D5, F, and N9a), which are known to be associated with differential risk of T2DM in both Koreans and Japanese, by adopting the cybrid model. We found that mitochondrial functions, as measured by ATP generation, ROS production, MMP, and cellular viability and dehydrogenase activity, did not vary among the three haplogroups. Previous studies have experimentally assessed functional differences caused by mtDNA haplogroups using cybrid models and reported mixed results. In one study, no bioenergetic differences were found between haplogroups H and T in terms of mitochondrial and cellular respiration rates, coupling efficiency, and MMP [26]. In another study, some functional differences were found between haplogroups H and Uk, such as MMP, growth capacity, and normalized oxygen consumption [27]. These inconsistent results suggest that some mtDNA haplogroups may be functionally neutral and others not. Alternatively, modest functional difference may be fully compensated by the nuclear genome, but under certain circumstances, this compensation may be not enough for maintaining normal cellular function.

Gene expression studies have demonstrated that the nuclear genome responds to impaired mitochondrial functions that arise from the depletion of mtDNA [28] or the mutation of mtDNA, such as A3243G [29]. Although we could not find robust functional differences in the mitochondrial function of cybrids, we investigated the genome-wide expression profile of cybrids by microarray to find possible compensatory mechanisms at the genomic level. Differential expression and cluster analyses revealed some evidence of nuclear genomic responses, which may be attributable to each mtDNA haplogroup tested in this study. First, even though an identical nuclear genomic background is shared among cybrids, pair-wise comparisons between cybrids with different mtDNA haplogroups exhibited a number of differentially expressed nuclear genes (Figure 3A), suggesting that nuclear gene expression is altered in response to possible differences in mitochondrial function related to different mtDNA haplogroups. Second, cybrids were clustered similarly according to mtDNA haplogroups when the cluster analysis was repeated using two types of expression profiles: the individual gene-level expression (Figure 3B) and the metabolic pathway-level expression (Figure 3C). Since the latter expression profile was derived solely from metabolic genes, the agreement of the two clustering analyses indicates that differences in metabolic transcriptome may be the main feature that distinguishes the three mtDNA haplogroups from one another.

It is noteworthy that the similarity in transcriptional responses elicited by different mtDNA haplogroups correlates with their reported susceptibility to T2DM. First, the number of differentially expressed genes was the smallest in the D5 vs. F comparison (Figure 3A), both of which are associated with an increased risk for T2DM (odds ratio 1.33 [95% CI 1.00–1.76] *p* = 0.0475, odds ratio 1.34 [95% CI 1.07–1.67] *p* = 0.0114, respectively [10]). However, when either D5 or F was compared to N9a, which is associated with a decreased risk for T2DM (odds ratio 0.55 [95% CI 0.40–0.75] *p* = 0.0002 [10]), more differential gene expression was observed than in the D5 vs. F comparison (Figure 3A). Second, haplogroups D5 and F were clustered together, whereas haplogroup N9a was separated from them by a large inter-cluster branch length (Figure 3B). Taken together, the possible alteration in mitochondrial function related to diabetes-susceptible mtDNA haplogroups (D5 and F) and the diabetes-resistant mtDNA haplogroup (N9a) may elicit distinct transcriptional responses in the nuclear genome. In this regard, it is worthy to note that mtDNA haplogroups N9a, F, and D are characterized by non-synonymous single nucleotide polymorphisms (SNPs): Thr8Ala in ND5 (N9a), Ser531Thr in ND5 (F), and Leu237Met in ND2 (D5) [10]. To assess the functional effect of the amino acid substitution shown above, we used the PolyPhen-2 program [30]. The substitutions observed in haplogroups F and D5 were both predicted as “possibly damaging” (i.e., affecting protein function): ND5 in haplogroup F with a score of 0.205 (sensitivity: 0.90; specificity: 0.83), and ND2 in haplogroup D5 with a score of 0.364 (sensitivity: 0.88; specificity: 0.85). The substitution at ND5 in haplogroup N9a did not yield a score since this substitution occurs at a position bearing no homology in the family of homologous proteins. Thus, the PolyPhen-2 analysis provides some supporting evidence that the substitutions observed at ND5 in haplogroup F and at ND2 in haplogroup D5 may be functional, whereas the substitution observed at ND5 in haplogroup N9a may be neutral.

By pathway analysis with GSEA, we identified several metabolic pathways that were differentially expressed between haplogroups (Table 1), including four pathways that were identified between haplogroups N9a and F. Notably, haplogroup F showed a decreased expression of the OXPHOS pathway and an increased expression of the glycolysis pathway. This observation can be regarded as a compensatory response for decreased ATP production caused by a defective mtDNA haplogroup, resulting in an increased expression of nuclear genes involved in glycolysis. GeneTrail analysis also exhibited a similar result. The pathway-level comparison of haplogroups N9a and F also revealed differential regulation of N-glycan biosynthesis and propanoate metabolism, where activities of both pathways were higher in N9a than in F. Further studies are required to examine the role of N-glycan biosynthesis and propanoate metabolism under abnormal mitochondrial function.

There are certain limitations in this study. We note that the apparent absence of differences in mitochondrial functions between the mtDNA haplogroups, as measured by biochemical experiments, might arise from the limited sample size of our study and the inability to detect very small functional changes. In addition, the use of the osteosarcoma-derived rho^0^ cell line prevented us from assessing mitochondrial functions in diabetes-relevant tissues, such as muscle, fat, liver, and pancreatic islets.

In summary, cybrids comprised of T2DM-associated mtDNA haplogroups did not show measurable differences in mitochondrial function. However, microarray analysis revealed differences in the expression of nuclear genes. Most notably, cybrids harboring the T2DM-susceptible F haplogroup exhibited significant differences in their gene expression profile at the pathway level compared to the T2DM-resistant N9a haplogroup. This suggests an important cross-talk between the mitochondria and the nucleus, where variation in mtDNA could affect expression of nuclear genes regulating mitochondrial function or cellular energetics. In this regard, we speculate that the defective nuclear compensation for impaired mitochondrial function caused by mtDNA factors may lead to functional alterations at the cellular level for certain nuclear and/or environmental factors (e.g., aging). Future studies are needed to identify the key signals mediating mitochondria-nuclear communication.

## Supporting Information

File S1 Lists of 210 differentially expressed genes that satisfied both the statistical significance criterion and pairwise fold change criterion >1.2-fold.
(XLS)

File S2 Detailed method of cell-based functional measurements.
(DOC)

Table S1 Genes that contribute the most to the differential regulation of metabolic pathways listed in Table 1. Genes that contribute the most to the differential regulation of metabolic pathways listed in Table 1.
(DOC)

Table S2 Significant differentially regulated metabolic pathways identified from haplogroup comparisons using GeneTrail.
(DOC)
