# Supplementary material for: Gene Expression Pattern in Transmitochondrial Cytoplasmic Hybrid Cells Harboring Type 2 Diabetes-Associated Mitochondrial DNA Haplogroups
Source: PLoS One. 2011 Jul 13;6(7):e22116. doi: 10.1371/journal.pone.0022116 (PMC3135611; doi:10.1371/journal.pone.0022116)
Supplement: File S2 — Detailed method of cell-based functional measurements. (DOC) [file pone.0022116.s002.doc]

Detailed method of cell-based functional measurements

For cell-based functional assays, cybrid cells were seeded in 96-well plates at a density of 1×10^6^ cells/ml. After stabilization for 24 hours at 37°C under 5% CO_2_/95% air (vol/vol), each functional assay was performed using Victor3 multilabel plate reader (PerkinElmer, Waltham, MA). The coefficient of variation for each assay was less than 15%.

Cellular ATP levels were measured using the ATPLite kit (PerkinElmer) according to the manufacturer’s instructions. After the culture medium was aspirated from plates, 100 µl of phosphate-buffered saline (PBS) was added to each well. A total of 50 µl of mammalian cell lysis solution, provided with the kit, was added to each well. The plate was put on an orbital shaker (700 rpm) for five minutes. A total of 50 µl of substrate solution was subsequently added to the wells, and the plate was put on an orbital shaker (700 rpm) for additional five minutes. After 10 minutes of dark adaptation, the luminescence was measured using Victor3 multilabel plate reader (PerkinElmer).

Reactive oxygen species (ROS) were measured using chloromethyl-2'7'-dichlorodihydrofluorescein diacetate acetyl ester (CM-H2DCFDA) (Sigma-Aldrich, St. Louis, MO). After the culture medium was aspirated from plates, 200 µl of 10 μg/ml CM-H2DCFDA was added to each well. Before fluorescence measurements, cells were incubated at 37°C for 10 minutes and washed with 200 µl of PBS per well three times. The Victor3 multilabel plate reader (PerkinElmer) was used to measure fluorescence at excitation/emission 485 nm/530 nm.

Mitochondrial membrane potential (MMP) was measured using 5,5’,6,6’-tetrachloro-1,1’,3,3’-tetraethylbenzimidazolylcarbocyanine iodide (JC-1) dye (Molecular Probes, Carlsbad, CA). The JC-1 dye changes its fluorescence according to MMP. When depolarized, it is converted to red fluorescent J-aggregates from the diffuse green form. The red to green fluorescence ratio serves as an indicator of MMP . After the culture medium was aspirated from plates, 200 µl of 2 µg/ml JC-1 was added to each well. Before fluorescence measurements, cells were incubated at 37°C for two hours and washed with 200 µl PBS per well five times. The Victor3 multilabel plate reader (PerkinElmer) was used to measure fluorescence at excitation/emission 530 nm/580 nm (red) and then at excitation/emission 485 nm/530 nm (green).

The cellular viability and mitochondrial dehydrogenase activity were assessed by the 3-(4,5-dimethylthiazol-2-yl)-2,5-diphenyltetrazolium bromide (MTT) (Sigma-Aldrich, St. Louis, MO) reduction assay. After the culture medium was aspirated from plates, 100 µl of 0.5 mg/ml MTT was added to each well. After incubation at 37°C for two hours, the MTT solution was aspirated and 100 µl of DMSO was added in each well to dissolve the formazan crystals. This was followed by incubation for 30 minutes at 37°C and an additional 30 minutes at room temperature. The Victor3 multilabel plate reader (PerkinElmer) was used to measure the absorbance at 540 nm.
