# Supplementary material for: Gene Expression Pattern in Transmitochondrial Cytoplasmic Hybrid Cells Harboring Type 2 Diabetes-Associated Mitochondrial DNA Haplogroups
Source: PLoS One. 2011 Jul 13;6(7):e22116. doi: 10.1371/journal.pone.0022116 (PMC3135611; doi:10.1371/journal.pone.0022116)
Supplement: Table S1 — Genes that contribute the most to the differential regulation of metabolic pathways listed in Table 1. Genes that contribute the most to the differential regulation of metabolic pathways listed in Table 1. (DOC) [file pone.0022116.s003.doc]

**Table S1.** Genes that contribute the most to the differential regulation of metabolic pathways listed in Table 1 of the main text. The genes are ranked by *t* statistic representing the extent of differential expression.

| Rank | Symbol | Gene name | | | | | | | |
| --- | --- | --- | --- | --- | --- | --- | --- | --- | --- |
| D5 vs. F | | N-Glycan biosynthesis (more active in D5) | | | |  | | | |
| 1 | ALG11 | asparagine-linked glycosylation 11 homolog (S. cerevisiae, alpha-1,2-mannosyltransferase) | | | | | | | |
| 2 | STT3B | STT3, subunit of the oligosaccharyltransferase complex, homolog B (S. cerevisiae) | | | | | | | |
| 3 | ALG14 | asparagine-linked glycosylation 14 homolog (yeast) | | | | | | | |
| 4 | ALG9 | asparagine-linked glycosylation 9 homolog (S. cerevisiae, alpha- 1,2-mannosyltransferase) | | | | | | | |
| 5 | ALG6 | asparagine-linked glycosylation 6 homolog (S. cerevisiae, alpha-1,3-glucosyltransferase) | | | | | | | |
| 6 | MGAT5 | mannosyl (alpha-1,6-)-glycoprotein beta-1,6-N-acetyl-glucosaminyltransferase | | | | | | | |
| 7 | DPAGT1 | dolichyl-phosphate (UDP-N-acetylglucosamine) N-acetylglucosaminephosphotransferase 1 (GlcNAc-1-P transferase) | | | | | | | |
| 8 | DHDDS | dehydrodolichyl diphosphate synthase | | | | | | | |
| 9 | ALG10 | asparagine-linked glycosylation 10 homolog (yeast, alpha-1,2-glucosyltransferase) | | | | | | | |
| 10 | ALG3 | asparagine-linked glycosylation 3 homolog (S. cerevisiae, alpha-1,3-mannosyltransferase) | | | | | | | |
| 11 | ALG8 | asparagine-linked glycosylation 8 homolog (S. cerevisiae, alpha-1,3-glucosyltransferase) | | | | | | | |
| 12 | GANAB | glucosidase, alpha; neutral AB | | | | | | | |
| 13 | ALG2 | asparagine-linked glycosylation 2 homolog (S. cerevisiae, alpha-1,3-mannosyltransferase) | | | | | | | |
| 14 | RFT1 | RFT1 homolog (S. cerevisiae) | | | | | | | |
| 15 | MAN1A1 | mannosidase, alpha, class 1A, member 1 | | | | | | | |
| 16 | MGAT1 | mannosyl (alpha-1,3-)-glycoprotein beta-1,2-N-acetylglucosaminyltransferase | | | | | | | |
| 17 | RPN2 | ribophorin II | | | | | | | |
| 18 | ALG1 | asparagine-linked glycosylation 1 homolog (S. cerevisiae, beta-1,4-mannosyltransferase) | | | | | | | |
| 19 | DOLPP1 | dolichyl pyrophosphate phosphatase 1 | | | | | | | |
| 20 | DPM1 | dolichyl-phosphate mannosyltransferase polypeptide 1, catalytic subunit | | | | | | | |
| 21 | DAD1 | defender against cell death 1 | | | | | | | |
| N9a vs. D5 | | Arachidonic acid metabolism (less active in N9a) | | | | | |  | |
| 1 | PTGES2 | prostaglandin E synthase 2 | | | | | | | |
| 2 | GPX2 | glutathione peroxidase 2 (gastrointestinal) | | | | | | | |
| 3 | CYP2C19 | cytochrome P450, family 2, subfamily C, polypeptide 19 | | | | | | | |
| 4 | GPX1 | glutathione peroxidase 1 | | | | | | | |
| 5 | ALOX5 | arachidonate 5-lipoxygenase | | | | | | | |
| 6 | PLA2G10 | phospholipase A2, group X | | | | | | | |
| 7 | CBR3 | carbonyl reductase 3 | | | | | | | |
| 8 | PLA2G2D | phospholipase A2, group IID | | | | | | | |
| 9 | ALOX15B | arachidonate 15-lipoxygenase, type B | | | | | | | |
| 10 | CYP2C9 | cytochrome P450, family 2, subfamily C, polypeptide 9 | | | | | | | |
| 11 | CYP4F3 | cytochrome P450, family 4, subfamily F, polypeptide 3 | | | | | | | |
| 12 | PLA2G2E | phospholipase A2, group IIE | | | | | | | |
| 13 | DHRS4 | dehydrogenase/reductase (SDR family) member 4 | | | | | | | |
| 14 | CYP4A22 | cytochrome P450, family 4, subfamily A, polypeptide 22 | | | | | | | |
| 15 | ALOX15 | arachidonate 15-lipoxygenase | | | | | | | |
| 16 | CYP2U1 | cytochrome P450, family 2, subfamily U, polypeptide 1 | | | | | | | |
| 17 | PLA2G3 | phospholipase A2, group III | | | | | | | |
| 18 | GPX5 | glutathione peroxidase 5 (epididymal androgen-related protein) | | | | | | | |
| 19 | TBXAS1 | thromboxane A synthase 1 (platelet, cytochrome P450, family 5, subfamily A) | | | | | | | |
| 20 | PLA2G12B | phospholipase A2, group XIIB | | | | | | | |
| 21 | PLA2G2A | phospholipase A2, group IIA (platelets, synovial fluid) | | | | | | | |
| 22 | PLA2G6 | phospholipase A2, group VI (cytosolic, calcium-independent) | | | | | | | |
| 23 | PTGIS | prostaglandin I2 (prostacyclin) synthase | | | | | | | |
| 24 | GPX7 | glutathione peroxidase 7 | | | | | | | |
| 25 | PTGS1 | prostaglandin-endoperoxide synthase 1 (prostaglandin G/H synthase and cyclooxygenase) | | | | | | | |
| N9a vs. F | | Glycolysis and gluconeogenesis (less active in N9a) | | | | | | |  |
| 1 | PFKL | phosphofructokinase, liver | | | | | | | |
| 2 | PKM2 | pyruvate kinase, muscle | | | | | | | |
| 3 | ALDOA | aldolase A, fructose-bisphosphate | | | | | | | |
| 4 | TPI1 | triosephosphate isomerase 1 | | | | | | | |
| 5 | ALDH3A1 | aldehyde dehydrogenase 3 family, memberA1 | | | | | | | |
| 6 | PGAM4 | phosphoglycerate mutase family member 4 | | | | | | | |
| 7 | ALDH3B1 | aldehyde dehydrogenase 3 family, member B1 | | | | | | | |
| 8 | PFKP | phosphofructokinase, platelet | | | | | | | |
| 9 | ACSS1 | acyl-CoA synthetase short-chain family member 1 | | | | | | | |
| 10 | HK1 | hexokinase 1 | | | | | | | |
| 11 | GPI | glucose phosphate isomerase | | | | | | | |
| 12 | PGAM1 | phosphoglycerate mutase 1 (brain) | | | | | | | |
| 13 | PGM1 | phosphoglucomutase 1 | | | | | | | |
| 14 | ENO3 | enolase 3 (beta, muscle) | | | | | | | |
| 15 | BPGM | 2,3-bisphosphoglycerate mutase | | | | | | | |
| 16 | GAPDHS | glyceraldehyde-3-phosphate dehydrogenase, spermatogenic | | | | | | | |
| 17 | ALDOB | aldolase B, fructose-bisphosphate | | | | | | | |
| 18 | HK2 | hexokinase 2 | | | | | | | |
| 19 | ACSS2 | acyl-CoA synthetase short-chain family member 2 | | | | | | | |
| 20 | FBP2 | fructose-1,6-bisphosphatase 2 | | | | | | | |
| 21 | ALDH3B2 | aldehyde dehydrogenase 3 family, member B2 | | | | | | | |
| 22 | ENO2 | enolase 2 (gamma, neuronal) | | | | | | | |
| 23 | ALDOC | aldolase C, fructose-bisphosphate | | | | | | | |
| N9a vs. F | | N-Glycan biosynthesis (more active in N9a) | |  | | | | | |
| 1 | ALG10 | asparagine-linked glycosylation 10 homolog (yeast, alpha-1,2-glucosyltransferase) | | | | | | | |
| 2 | STT3B | STT3, subunit of the oligosaccharyltransferase complex, homolog B (S. cerevisiae) | | | | | | | |
| 3 | ALG6 | asparagine-linked glycosylation 6 homolog (S. cerevisiae, alpha-1,3-glucosyltransferase) | | | | | | | |
| 4 | MAN1A2 | mannosidase, alpha, class 1A, member 2 | | | | | | | |
| 5 | ALG10B | asparagine-linked glycosylation 10 homolog B (yeast, alpha-1,2-glucosyltransferase) | | | | | | | |
| 6 | MAN1A1 | mannosidase, alpha, class 1A, member 1 | | | | | | | |
| 7 | ALG11 | asparagine-linked glycosylation 11 homolog (S. cerevisiae, alpha-1,2-mannosyltransferase) | | | | | | | |
| 8 | DPM1 | dolichyl-phosphate mannosyltransferase polypeptide 1, catalytic subunit | | | | | | | |
| 9 | ALG13 | asparagine-linked glycosylation 13 homolog (S. cerevisiae) | | | | | | | |
| 10 | MGAT5 | mannosyl (alpha-1,6-)-glycoprotein beta-1,6-N-acetyl-glucosaminyltransferase | | | | | | | |
| 11 | FUT8 | fucosyltransferase 8 (alpha (1,6) fucosyltransferase) | | | | | | | |
| 12 | ALG2 | asparagine-linked glycosylation 2 homolog (S. cerevisiae, alpha-1,3-mannosyltransferase) | | | | | | | |
| 13 | ALG9 | asparagine-linked glycosylation 9 homolog (S. cerevisiae, alpha- 1,2-mannosyltransferase) | | | | | | | |
| 14 | ALG14 | asparagine-linked glycosylation 14 homolog (yeast) | | | | | | | |
| 15 | MAN2A1 | mannosidase, alpha, class 2A, member 1 | | | | | | | |
| 16 | ALG8 | asparagine-linked glycosylation 8 homolog (S. cerevisiae, alpha-1,3-glucosyltransferase) | | | | | | | |
| 17 | RPN2 | ribophorin II | | | | | | | |
| 18 | RFT1 | RFT1 homolog (S. cerevisiae) | | | | | | | |
| 19 | ALG5 | asparagine-linked glycosylation 5 homolog (S. cerevisiae, dolichyl-phosphate beta-glucosyltransferase) | | | | | | | |
| 20 | DAD1 | defender against cell death 1 | | | | | | | |
| 21 | ALG3 | asparagine-linked glycosylation 3 homolog (S. cerevisiae, alpha-1,3-mannosyltransferase) | | | | | | | |
| 22 | DHDDS | dehydrodolichyl diphosphate synthase | | | | | | | |
| N9a vs. F | | Propanoate metabolism (more active in N9a) | | |  | | | | |
| 1 | ACADM | acyl-Coenzyme A dehydrogenase, C-4 to C-12 straight chain | | | | | | | |
| 2 | SUCLA2 | succinate-CoA ligase, ADP-forming, beta subunit | | | | | | | |
| 3 | SUCLG1 | succinate-CoA ligase, GDP-forming, alpha subunit | | | | | | | |
| 4 | LDHB | lactate dehydrogenase B | | | | | | | |
| 5 | ALDH3A2 | aldehyde dehydrogenase 3 family, member A2 | | | | | | | |
| 6 | PCCA | propionyl Coenzyme A carboxylase, alpha polypeptide | | | | | | | |
| 7 | ALDH9A1 | aldehyde dehydrogenase 9 family, member A1 | | | | | | | |
| 8 | HIBCH | 3-hydroxyisobutyryl-Coenzyme A hydrolase | | | | | | | |
| 9 | ACAT1 | acetyl-Coenzyme A acetyltransferase 1 (acetoacetyl Coenzyme A thiolase) | | | | | | | |
| 10 | MCEE | methylmalonyl CoA epimerase | | | | | | | |
| 11 | ALDH6A1 | aldehyde dehydrogenase 6 family, member A1 | | | | | | | |
| 12 | ACACA | acetyl-Coenzyme A carboxylase alpha | | | | | | | |
| 13 | HADHA | hydroxyacyl-Coenzyme A dehydrogenase/3-ketoacyl-Coenzyme A thiolase/enoyl-Coenzyme A hydratase (trifunctional protein), alpha subunit | | | | | | | |
| 14 | MLYCD | malonyl-CoA decarboxylase | | | | | | | |
| 15 | ACAT2 | acetyl-Coenzyme A acetyltransferase 2 (acetoacetyl Coenzyme A thiolase) | | | | | | | |
| 16 | EHHADH | enoyl-Coenzyme A, hydratase/3-hydroxyacyl Coenzyme A dehydrogenase | | | | | | | |
| 17 | LDHAL6A | lactate dehydrogenase A-like 6A | | | | | | | |
| 18 | LDHC | lactate dehydrogenase C | | | | | | | |
| 19 | ALDH2 | aldehyde dehydrogenase 2 family (mitochondrial) | | | | | | | |
| 20 | MUT | methylmalonyl Coenzyme A mutase | | | | | | | |
| N9a vs. F | | Oxidative phosphorylation (more active in N9a) | | | | |  | | |
|  | | Activated components |  | | | | | | |
| 1 | NDUFB5 | NADH dehydrogenase (ubiquinone) 1 beta subcomplex, 5, 16kDa | | | | | | | |
| 2 | ATP6V1G1 | ATPase, H+ transporting, lysosomal 13kDa, V1 subunit G1 | | | | | | | |
| 3 | ATP6V0A1 | ATPase, H+ transporting, lysosomal V0 subunit a1 | | | | | | | |
| 4 | ATP5O | ATP synthase, H+ transporting, mitochondrial F1 complex, O subunit (oligomycin sensitivity conferring protein) | | | | | | | |
| 5 | COX6C | cytochrome c oxidase subunit VIc | | | | | | | |
| 6 | ATP5L | ATP synthase, H+ transporting, mitochondrial F0 complex, subunit G | | | | | | | |
| 7 | NDUFB9 | NADH dehydrogenase (ubiquinone) 1 beta subcomplex, 9, 22kDa | | | | | | | |
| 8 | ND3 | mitochondrially encoded NADH dehydrogenase 3 | | | | | | | |
| 9 | ATP6V1C1 | ATPase, H+ transporting, lysosomal 42kDa, V1 subunit C1 | | | | | | | |
| 10 | NDUFC1 | NADH dehydrogenase (ubiquinone) 1, subcomplex unknown, 1, 6kDa | | | | | | | |
| 11 | COX7C | cytochrome c oxidase subunit VIIc | | | | | | | |
| 12 | PPA1 | pyrophosphatase (inorganic) 1 | | | | | | | |
| 13 | NDUFA5 | NADH dehydrogenase (ubiquinone) 1 alpha subcomplex, 5, 13kDa | | | | | | | |
| 14 | ATP5F1 | ATP synthase, H+ transporting, mitochondrial F0 complex, subunit B1 | | | | | | | |
| 15 | NDUFC2 | NADH dehydrogenase (ubiquinone) 1, subcomplex unknown, 2, 14.5kDa | | | | | | | |
| 16 | ATP6V1H | ATPase, H+ transporting, lysosomal 50/57kDa, V1 subunit H | | | | | | | |
| 17 | NDUFS1 | NADH dehydrogenase (ubiquinone) Fe-S protein 1, 75kDa (NADH-coenzyme Q reductase) | | | | | | | |
| 18 | ATP5J | ATP synthase, H+ transporting, mitochondrial F0 complex, subunit F6 | | | | | | | |
| 19 | ATP5C1 | ATP synthase, H+ transporting, mitochondrial F1 complex, gamma polypeptide 1 | | | | | | | |
| 20 | ATP6V1C2 | ATPase, H+ transporting, lysosomal 42kDa, V1 subunit C2 | | | | | | | |
| 21 | COX15 | COX15 homolog, cytochrome c oxidase assembly protein (yeast) | | | | | | | |
| 22 | ATP5G3 | ATP synthase, H+ transporting, mitochondrial F0 complex, subunit C3 (subunit 9) | | | | | | | |
| 23 | ATP5G1 | ATP synthase, H+ transporting, mitochondrial F0 complex, subunit C1 (subunit 9) | | | | | | | |
| 24 | ATP6V0B | ATPase, H+ transporting, lysosomal 21kDa, V0 subunit b | | | | | | | |
| 25 | NDUFB6 | NADH dehydrogenase (ubiquinone) 1 beta subcomplex, 6, 17kDa | | | | | | | |
| 26 | UQCRB | ubiquinol-cytochrome c reductase binding protein | | | | | | | |
| 27 | NDUFS2 | NADH dehydrogenase (ubiquinone) Fe-S protein 2, 49kDa (NADH-coenzyme Q reductase) | | | | | | | |
| 28 | NDUFA11 | NADH dehydrogenase (ubiquinone) 1 alpha subcomplex, 11, 14.7kDa | | | | | | | |
| 29 | UCRC | ubiquinol-cytochrome c reductase, complex III subunit X | | | | | | | |
| 30 | ATP6V1A | ATPase, H+ transporting, lysosomal 70kDa, V1 subunit A | | | | | | | |
| 31 | ATP6V1G2 | ATPase, H+ transporting, lysosomal 13kDa, V1 subunit G2 | | | | | | | |
| 32 | NDUFS6 | NADH dehydrogenase (ubiquinone) Fe-S protein 6, 13kDa (NADH-coenzyme Q reductase) | | | | | | | |
| 33 | NDUFAB1 | NADH dehydrogenase (ubiquinone) 1, alpha/beta subcomplex, 1, 8kDa | | | | | | | |
| 34 | ATP5D | ATP synthase, H+ transporting, mitochondrial F1 complex, delta subunit | | | | | | | |
| 35 | ATP6V1B2 | ATPase, H+ transporting, lysosomal 56/58kDa, V1 subunit B2 | | | | | | | |
| 36 | ATP6V1E1 | ATPase, H+ transporting, lysosomal 31kDa, V1 subunit E1 | | | | | | | |
| 37 | ND2 | mitochondrially encoded NADH dehydrogenase 2 | | | | | | | |
| 38 | PPA2 | pyrophosphatase (inorganic) 2 | | | | | | | |
| 39 | COX8C | cytochrome c oxidase subunit 8C | | | | | | | |
| 40 | ND6 | mitochondrially encoded NADH dehydrogenase 6 | | | | | | | |
| 41 | UQCRC2 | ubiquinol-cytochrome c reductase core protein II | | | | | | | |
| 42 | NDUFA12 | NADH dehydrogenase (ubiquinone) 1 alpha subcomplex, 12 | | | | | | | |
| 43 | ATP5H | ATP synthase, H+ transporting, mitochondrial F0 complex, subunit d | | | | | | | |
| 44 | COX3 | mitochondrially encoded cytochrome c oxidase III | | | | | | | |
| 45 | ATP5G2 | ATP synthase, H+ transporting, mitochondrial F0 complex, subunit C2 (subunit 9) | | | | | | | |
|  | | Repressed components |  | | | | | | |
| 1 | ATP12A | ATPase, H+/K+ transporting, nongastric, alpha polypeptide | | | | | | | |
| 2 | UQCRC1 | ubiquinol-cytochrome c reductase core protein I | | | | | | | |
| 3 | SDHA | succinate dehydrogenase complex, subunit A, flavoprotein (Fp) | | | | | | | |
| 4 | CYC1 | cytochrome c-1 | | | | | | | |
| 5 | TCIRG1 | T-cell, immune regulator 1, ATPase, H+ transporting, lysosomal V0 subunit A3 | | | | | | | |
| 6 | ATP6V1E2 | ATPase, H+ transporting, lysosomal 31kDa, V1 subunit E2 | | | | | | | |
| 7 | COX6A1 | cytochrome c oxidase subunit VIa polypeptide 1 | | | | | | | |
| 8 | NDUFA13 | NADH dehydrogenase (ubiquinone) 1 alpha subcomplex, 13 | | | | | | | |
| 9 | COX4I2 | cytochrome c oxidase subunit IV isoform 2 (lung) | | | | | | | |
| 10 | NDUFA4L2 | NADH dehydrogenase (ubiquinone) 1 alpha subcomplex, 4-like 2 | | | | | | | |
| 11 | NDUFS5 | NADH dehydrogenase (ubiquinone) Fe-S protein 5, 15kDa (NADH-coenzyme Q reductase) | | | | | | | |
| 12 | COX6B1 | cytochrome c oxidase subunit Vib polypeptide 1 (ubiquitous) | | | | | | | |
| 13 | NDUFB8 | NADH dehydrogenase (ubiquinone) 1 beta subcomplex, 8, 19kDa | | | | | | | |
| 14 | NDUFV1 | NADH dehydrogenase (ubiquinone) flavoprotein 1, 51kDa | | | | | | | |
| 15 | NDUFS8 | NADH dehydrogenase (ubiquinone) Fe-S protein 8, 23kDa (NADH-coenzyme Q reductase) | | | | | | | |
| 16 | UQCR | ubiquinol-cytochrome c reductase, 6.4kDa subunit | | | | | | | |
