# Supplementary material for: Gene Expression Pattern in Transmitochondrial Cytoplasmic Hybrid Cells Harboring Type 2 Diabetes-Associated Mitochondrial DNA Haplogroups
Source: PLoS One. 2011 Jul 13;6(7):e22116. doi: 10.1371/journal.pone.0022116 (PMC3135611; doi:10.1371/journal.pone.0022116)
Supplement: Table S2 — Significant differentially regulated metabolic pathways identified from haplogroup comparisons using GeneTrail. (DOC) [file pone.0022116.s004.doc]

**Table S2.** Significant differentially regulated metabolic pathways identified from haplogroup comparisons using GeneTrail (raw *p*-value <0.05 and FDR <0.25). Abbreviations: FDR—false discovery rate.

| Comparison | Pathway ID | Pathway name | Raw  *p*-value | FDR |
| --- | --- | --- | --- | --- |
| D5 vs. F | Less active in D5 (More active in F) | |  |  |
|  | HSA00030 | Pentose phosphate pathway | 0.003960 | 0.056760 |
|  | HSA00251 | Glutamate metabolism | 0.045951 | 0.193907 |
|  | More active in D5 (Less active in F) | |  |  |
|  | HSA00510 | N-Glycan biosynthesis | 8.4E-06 | 0.000361 |
|  | HSA00190 | Oxidative phosphorylation | 0.001313 | 0.028229 |
|  | HSA00120 | Bile acid biosynthesis | 0.008244 | 0.088625 |
|  | HSA00240 | Pyrimidine metabolism | 0.017706 | 0.152271 |
|  | HSA00561 | Glycerolipid metabolism | 0.023841 | 0.170857 |
|  | HSA00512 | O-Glycan biosynthesis | 0.031025 | 0.190583 |
|  | HSA00280 | Valine, leucine and isoleucine degradation | 0.044239 | 0.193907 |
|  | HSA00650 | Butanoate metabolism | 0.046524 | 0.193907 |
| N9a vs. D5 | Less active in N9a (More active in D5) | |  |  |
|  | HSA00051 | Fructose and mannose metabolism | 0.000399 | 0.015377 |
|  | HSA00590 | Arachidonic acid metabolism | 0.000715 | 0.015377 |
|  | HSA00591 | Linoleic acid metabolism | 0.014065 | 0.120956 |
|  | HSA00030 | Pentose phosphate pathway | 0.017229 | 0.123475 |
|  | HSA00010 | Glycolysis and gluconeogenesis | 0.026523 | 0.126979 |
|  | HSA00052 | Galactose metabolism | 0.026577 | 0.126979 |
|  | More active in N9a (Less active in D5) | |  |  |
|  | HSA00640 | Propanoate metabolism | 0.000400 | 0.057317 |
|  | HSA00280 | Valine, leucine and isoleucine degradation | 0.010665 | 0.114650 |
|  | HSA00510 | N-Glycan biosynthesis | 0.022798 | 0.126979 |
| N9a vs. F | Less active in N9a (More active in F) | |  |  |
|  | HSA00030 | Pentose phosphate pathway | 2.5E-05 | 0.000266 |
|  | HSA00051 | Fructose and mannose metabolism | 0.000399 | 0.003431 |
|  | HSA00052 | Galactose metabolism | 0.007646 | 0.046967 |
|  | HSA00480 | Glutathione metabolism | 0.015663 | 0.067555 |
|  | HSA00010 | Glycolysis and gluconeogenesis | 0.034817 | 0.105811 |
|  | HSA00590 | Arachidonic acid metabolism | 0.036911 | 0.105811 |
|  | More active in N9a (Less active in F) | |  |  |
|  | HSA00640 | Propanoate metabolism | 1.0E-05 | 0.000266 |
|  | HSA00510 | N-Glycan biosynthesis | 1.5E-05 | 0.000266 |
|  | HSA00190 | Oxidative phosphorylation | 2.1E-05 | 0.000266 |
|  | HSA00280 | Valine, leucine and isoleucine degradation | 0.001919 | 0.013750 |
|  | HSA00240 | Pyrimidine metabolism | 0.011671 | 0.062733 |
|  | HSA00410 | Beta alanine metabolism | 0.016682 | 0.067555 |
|  | HSA00071 | Fatty acid metabolism | 0.017282 | 0.067555 |
|  | HSA00790 | Folate biosynthesis | 0.028944 | 0.101087 |
|  | HSA00120 | Bile acid biosynthesis | 0.030561 | 0.101087 |
